# Correlation models for monitoring fetal growth

**DOI:** 10.1177/0962280220905623

**Published:** 2020-03-23

**Authors:** Yuan Feng, Luo Xiao, Cai Li, Stephanie T Chen, Eric O Ohuma

**Affiliations:** 1 Department of Statistics, North Carolina State University, Raleigh, NC, USA; 2 Centre for Tropical Medicine and Global Health, Nuffield Department of Medicine, University of Oxford, Oxford, UK; 3 Nuffield Department of Women’s & Reproductive Health, University of Oxford, John Radcliffe Hospital, Headington, Oxford, UK

**Keywords:** Fetal health, longitudinal study, correlation, reference chart

## Abstract

Ultrasound growth measurements are monitored to evaluate if a fetus is growing normally compared with a defined standard chart at a specified gestational age. Using data from the Fetal Growth Longitudinal Study of the INTERGROWTH-21^st^ project, we have modelled the longitudinal dependence of fetal head circumference, biparietal diameter, occipito-frontal diameter, abdominal circumference, and femur length using a two-stage approach. The first stage involved finding a suitable transformation of the raw fetal measurements (as the marginal distributions of ultrasound measurements were non-normal) to standardized deviations (Z-scores). In the second stage, a correlation model for a Gaussian process is fitted, yielding a correlation for any pair of observations made between 14 and 40 weeks. The correlation structure of the fetal Z-score can be used to assess whether the growth, for example, between successive measurements is satisfactory. The paper is accompanied by a Shiny application, see https://lxiao5.shinyapps.io/shinycalculator/.

## 1 Introduction

During pregnancy, fetal anthropometric measures consisting of head circumference (HC), biparietal diameter (BPD), occipito-frontal diameter (OFD), abdominal circumference (AC), and femur length (FL) are measured using ultrasound to monitor attained fetal size at a given gestational age (GA). By comparing measurements to a reference or standard chart,^1,2^ fetuses with measurements at the tails of the distribution (for example below the 3^rd^, 5^th^, or 10^th^ centiles or above the 90^th^, 95^th^, or 97^th^ centiles) are identified as being at increased risk of a growth disorder, such as intra-uterine growth restriction (IUGR) that may require further investigation. Growth charts, which conventionally record only cross-sectional (attained size) information, can be extended to monitor growth rate over time (velocity).^3^ An assessment of the current size of the fetus in relation to the size in the past (the previous visit) enables the evaluation of an individual’s growth between any two time points (rate of growth). These changes observed between two time points may be used to identify those requiring closer monitoring. Fetal growth is rapid in the first and second trimester and slows towards term. The correlation of measurements from the same fetus is important for evaluating fetal growth velocity. The correlation coefficient is not constant as it is dependent on the interval between measurements. An estimate of the correlation coefficient is straightforward for fixed time intervals, but it is clinically useless as, in normal practice, fetuses are seen and measured at irregularly spaced time points – a model that allows for such irregularity is required. Correlation models have previously been derived for child data^4–8^ but not for fetal biometry data.

We model the correlation of fetal biometry (i.e. HC, BPD, OFD, AC, and FL) and derive formulae and a Shiny application that can be used to obtain the correlation for each fetal measure between measurements made at any two time points between 14 and 40 weeks of GA. We model the correlations using fetal ultrasound data from the INTERGROWTH-21^st^ Project Fetal Growth Longitudinal Study (FGLS) on which the international standards for fetal growth are based.^9,10^ A separate analysis of the cohort demonstrated that the FGLS cohort remained healthy with adequate growth and motor development up to 2 years of age.^11^

## 2 Data

The INTERGROWTH-21^st^ Project was a population-based longitudinal study that measured serial fetal growth scans every 5^±1 ^weeks from recruitment at 9^+0^ – 13^+6 ^weeks of gestation until, but not beyond, 42^+0 ^weeks of gestation. The FGLS component of the INTERGROWTH-21^st^ Project is the largest prospective study to collect data on fetal ultrasound measurements from optimally healthy pregnant women to date, collecting data in eight geographically diverse populations and using many quality control measures. The FGLS involved measuring serial fetal growth scans every 5^±1 ^weeks after the initial dating scan, so that the possible ranges after the dating scan were 14–18, 19–23, 24–28, 29–33, 34–38, and 39–42 weeks of gestation. To ensure that all sites collected high-quality data that were comparable within and between the study sites, all sonographers and anthropometrists were trained, and all ultrasound measurements were performed in a standardized manner following strict protocols.^12^ All sites adopted uniform methods, used identical ultrasound equipment in all of the study sites, adopted standardized methodology to take fetal measurements, and employed locally accredited ultra-sonographers who underwent standardization training and monitoring.

**Figure 1.**
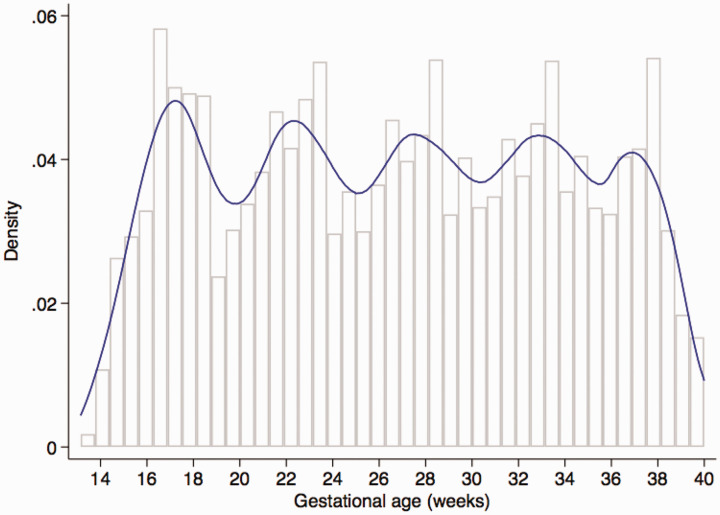
Distribution of Gestational Age at which measurements were recorded (with expected periodicity of 5 weeks).

The FGLS screened 13,108 pregnant women attending the study clinics $<14^{+0}$ weeks of gestation within the project’s defined geographical areas; of these, 4607 (35%) who met the eligibility criteria gave informed consent and enrolled. The most common reasons for ineligibility were low maternal height (13%), BMI ≥ 30 (12%), and maternal age < 18 or > 35 years (11%). Thirty-six women (0.8%) who developed severe conditions during pregnancy or took up smoking or used drugs, and 71 (1.5%) who were lost to follow-up or withdrew consent were excluded. A total of 4422 women delivered a live singleton, of which 4321 women (20,313 ultrasound scans) who had pregnancies without major complications and delivered live singletons without congenital malformations that contributed data for the construction of the INTERGROWTH-21^st^ international fetal growth standards,^9^ international gestation-specific newborn standards,^13^ gestational weight gain standards,^14^ and preterm postnatal growth standards^15^ were used for the present analysis. This cohort experienced very low maternal and perinatal mortality and morbidity rates,^9,13^ confirming that the participants were at low risk of adverse outcome and therefore contributed to the construction of the international fetal growth standards. The baseline characteristics of the study cohort across the eight sites were very similar, which was expected because women were selected from the underlying low-risk populations using the same clinical and demographic criteria.^9,16^ The median number of ultrasound scans (excluding the dating scan) was 5.0 (mean = 4.9, SD = 0.8, range from 4 to 7) and 97% of women had four scans. Eighty-five percent of the 20,313 ultrasound scans were performed within the expected gestational age window of the protocol as shown in Figure 1.^9^

The INTERGROWTH-21^st^ Project was approved by the Oxfordshire Research Ethics Committee “C” (reference: 08/H0606/139), the research ethics committees of the individual participating institutions, and the corresponding regional health authorities where the project was implemented. Participants provided written consent to be involved in the study.

## 3 Statistical methodology

Consider the longitudinal data $\left\{(T_{ij},Y_{ij}),1\leq j\leq m_{i},1\leq i\leq n\right\}$, where *T_ij_* is the gestational age in weeks for subject *i* at the *j*th visit, *Y_ij_* is one of the five ultrasound growth measurements in millimeters at *T_ij_*, *m_i_* is the number of visits for subject *i*, and *n* is the number of subjects. The total number of visits per woman is shown in Table 1.

**Table 1.** Summary of the number of women at each visit and the total number of follow-up visits.

No. of follow up visits (X)	No. who visited only X times (%)	No. who visited at least X times (%)
1	39 (0.9)	4233 (100.0)
2	55 (1.3)	4194 (99.1)
3	203 (4.8)	4139 (97.8)
4	810 (19.1)	3936 (93.0)
5	2724 (64.4)	3126 (73.8)
6	402 (9.5)	402 (9.5)
Total	4233 (100.0)	20 030 (100.0)

We estimate a correlation matrix of the ultrasound measurement at different gestational ages. A single model is fitted for both sexes as some mothers do not want to know the sex of the child they expect. Because the marginal distributions of ultrasound growth measurements may be non-normal, e.g. skewed, a suitable transformation of the raw growth measurements is first identified and applied to the data to construct a working marginal reference chart. The raw fetal measurements are then transformed accordingly to provide standardized deviations (Z-scores). Next the Z-scores are modeled by a Gaussian process with zero mean and unit variance so that the temporal correlation of the process can be estimated.

### 3.1 Working models for marginal reference distribution

We consider the LMS transformation^17^ which could transform non-normal data to make the assumption of normality acceptable. Let *Y* be a positive random variable and its LMS transformation is given by

(1) $$Z=\{\begin{array}{l} \frac{1}{\sigma \nu }\left\{{\left(\frac{Y}{\mu }\right)}^{\nu }-1\right\}\text{if }\;\nu \neq 0,\\ \frac{1}{\sigma }\log\left(\frac{Y}{\mu }\right)\text{if }\;\nu =0 \end{array}$$

Here $\mu ,\sigma \in {\mathbb{R}}^{+}$ and ν∈ℝ are location, scale and skewness parameters, respectively. If *Z* has a standard normal distribution, then *Y* is said to follow the three-parameter Box-Cox Cole-Green distribution^17^ denoted by BCCG(μ,σ,ν). A fourth parameter can be added to further model kurtosis: if *Z* has a *t* distribution with degrees of freedom $\tau \in {\mathbb{R}}^{+}$, then *Y* is said to follow the Box-Cox *t* distribution^18^ denoted by BCT(μ,σ,ν,τ); if *Z* has a standard power exponential distribution with parameter $\tau \in {\mathbb{R}}^{+}$, then *Y* is said to follow the Box-Cox power exponential distribution^19^ denoted by BCPE(μ,σ,ν,τ). Note that BCT(μ,σ,ν,τ=+∞) and BCPE(μ,σ,ν,τ=2) reduce to BCCG(μ,σ,ν).

We model the parameters in equation (1), μ(t), ν(t) and σ(t) as a smooth function of gestational age in conjunction with BCCG. The additional parameters in BCT and BCPE are defined similarly. The GAMLSS method^20^ can be used to estimate such functions. Under the LMS framework, suppose that $\left\{\hat{\mu }(t),\hat{\sigma }(t),\hat{\nu }(t)\right\}$ are the obtained estimates, then the transformed measurements *Z_ij_* can be computed as

(2) $$Z_{ij}=\{\begin{array}{l} \frac{1}{\hat{\sigma }(T_{ij})\hat{\nu }(T_{ij})}\left\{{\left(\frac{Y_{ij}}{\hat{\mu }(T_{ij})}\right)}^{\hat{\nu }(T_{ij})}-1\right\}\text{if }\;\hat{\nu }(T_{ij})\neq 0,\\ \frac{1}{\hat{\sigma }(T_{ij})}\log\left(\frac{Y_{ij}}{\hat{\mu }(T_{ij})}\right)\text{if }\;\hat{\nu }(T_{ij})=0 \end{array}$$

Under the BCCG model, marginally *Z_ij_* has approximately a standard normal distribution. Under the BCT or the BCPE models, additional transforms of *Z_ij_* are needed to make *Z_ij_* normal. For simplicity, we assume all proper transformations have been applied. The Gaussian process is fully identified by its correlation matrix, which we estimate with zero mean and unit variance.

### 3.2 Correlation models

In this section, we estimate a correlation matrix for the Z-scores. We compare several parametric and nonparametric models. The parametric models considered here have been applied to child growth. The exponential model^21^ (denoted by P1) is

$$\text{cor}(Z_{ij},Z_{ik})=\exp\left\{-b|T_{ij}-T_{ik}{|}^{a}\right\}$$

where $a,b\in {\mathbb{R}}^{+}$ are two unknown parameters that can be interpreted as the order and the rate of the change in the correlation. This model is commonly used due to its simple form and stationarity, i.e. the correlation depends only on the distance between two gestational ages. The second model (denoted by P2), proposed by^4^ for child growth, takes the form

$$\text{cor}(Z_{ij},Z_{ik})=\exp\left\{-b\left|\log\frac{T_{ij}+\tau }{T_{ik}+\tau }\right|\right\}$$

where $\tau ,b\in {\mathbb{R}}^{+}$ are two unknown parameters. The model is non-stationary, but possesses the Markovian property. Indeed, via the transformation $S_{ij}=\log(T_{ij}+\tau )$ and $S_{ik}=\log(T_{ik}+\tau )$, the correlation becomes $\text{cor}(Z_{ij},Z_{ik})=\rho^{\left|S_{ij}-S_{ik}\right|},$ where ρ=exp⁡(−b). Because growth measurements might have non-ignorable measurement errors, a nugget effect term is usually added to the above correlation models. The exponential correlation with a nugget effect model (denoted by P1+) takes the form

$$\text{cor}(Z_{ij},Z_{ik})=\frac{1}{1+\sigma^{2}}\left[\exp\left\{-b|T_{ij}-T_{ik}{|}^{a}\right\}+\sigma^{2}{\mathrm{11}}_{\left\{T_{ij}=T_{ik}\right\}}\right]$$

where $\sigma^{2}$ is the variance of the measurement error in the Z-scores and $11_{\left\{\right\}}$ is the indicator function which is 1 if the statement inside the bracket is true and 0 otherwise. Similarly, the P2+ correlation model has the form

$$\text{cor}(Z_{ij},Z_{ik})=\frac{1}{1+\sigma^{2}}\left[\exp\left\{-b\left|\log\frac{T_{ij}+\tau }{T_{ik}+\tau }\right|\right\}+\sigma^{2}\;11_{\left\{T_{ij}=T_{ik}\right\}}\right]$$

Note that with the nugget term, neither the stationary property nor the Markovian property holds.

Parametric models are simple and easy to interpret, but they can be subject to model misspecification. Thus, in addition to the above parametric correlation models, we also considered two nonparametric correlation models. The first one is based on functional data analysis,^22^ which models the Z-score of a subject as the sum of a smooth random function of the gestational age and a random measurement error term. Specifically, the functional data model is

(3) $$Z_{ij}=b_{i}(T_{ij})+\epsilon_{ij}$$

where $b_{i}(\cdot )$ is a smooth random function modeled by a zero-mean Gaussian process with a smooth covariance function $C(T_{ij},T_{ik})=\text{Cov}\{b_{i}(T_{ij}),b_{i}(T_{ik})\}$, $\left\{\epsilon_{i1},\ldots ,\epsilon_{im_{i}}\right\}$ are independent measurement errors with variance $\sigma_{\epsilon }^{2}$, and $b_{i}(\cdot )$ is independent from the measurement errors. Such a covariance function involves no parametric assumptions. Since $\text{Var}(Z_{ij})=1$, the correlation of the Z-scores at two distinct time points *T_ij_* and *T_ik_* with j≠k is $C(T_{ij},T_{ik})$. Estimation of this correlation matrix is described in Section 3.3.

The correlation function from the functional data method is in general nonstationary. We also consider a stationary but nonparametric correlation function by assuming that the correlation function C satisfies $C(T_{ij},T_{ik})=g(|T_{ij}-T_{ik}|)$, where *g* is a smooth decreasing but unspecified univariate function. Due to the presence of measurement error in the functional data model, the overall correlation between the Z-scores is still nonstationary. The estimation of *g* is addressed in Section 3.3. The various correlation models are summarized in Table 2.

**Table 2.** Correlation models.

Model	Abbreviation	Correlation form
Exponential	P1	$\exp\left\{-b|T_{ij}-T_{ik}{|}^{a}\right\}$
Exponential with nugget effect	P1+	$\frac{1}{1+\sigma^{2}}\left[\exp\left\{-b|T_{ij}-T_{ik}{|}^{a}\right\}+\sigma^{2}\left\{T_{ij}=T_{ik}\right\}\right]$
Markovian	P2	$\exp\left\{-b\left|\log\frac{T_{ij}+\tau }{T_{ik}+\tau }\right|\right\}$
Markovian with nugget effect	P2+	$\frac{1}{1+\sigma^{2}}\left[\exp\left\{-b\left|\log\frac{T_{ij}+\tau }{T_{ik}+\tau }\right|\right\}+\sigma^{2}\left\{T_{ij}=T_{ik}\right\}\right]$
1st nonparametric	NP1	$C(T_{ij},T_{ik})$: fully unspecified and smooth
2nd nonparametric	NP2	$C(T_{ij},T_{ik})=g(|T_{ij}-T_{ik}|)$: *g* unspecified and smooth

### 3.3 Estimation of the correlation models

The parametric correlation models are fitted by maximizing likelihood of the *Z*-scores under normality. We now focus on the estimation of the two nonparametric models. Estimation methods for the functional data model are well developed in the statistics literature and here we use the fast covariance estimation method for longitudinal data, developed in Xiao et al.^23^ We briefly describe the method here, which will also be useful for explaining our estimation method for NP2. First, empirical estimates of the correlation function are constructed. Specifically, let $r_{\mathrm{ijk}}=Z_{ij}Z_{ik}$ for $1\leq j,k\leq m_{i},1\leq i\leq n$. Then $E(r_{\mathrm{ijk}})=C(T_{ij},T_{ik})+11_{\left\{T_{ij}=T_{ik}\right\}}\sigma_{\epsilon }^{2}$. Thus, *r_ijk_* is an unbiased estimate of $C(T_{ij},T_{ik})$ whenever j≠k. We will conduct a bivariate smoothing of the data $\left\{(T_{ij},T_{ik},r_{\mathrm{ijk}}),1\leq j\neq k\leq m_{i},1\leq i\leq n\right\}$ to estimate the correlation function C. We use bivariate *P*-splines,^24^ which approximate the bivariate correlation function with tensor-product B-splines and employ a penalty to avoid overfit. The penalty ensures the smoothness of the fitted correlation function, a desirable feature for fetal growth correlations. Moreover, constraints on spline coefficients are imposed to ensure that C is symmetric; see Xiao et al.^23^ for further details. Denote the corresponding estimate by $\hat{C}(s,t)$, then we estimate the error variance $\sigma_{\epsilon }^{2}$ using the identity $E(r_{\mathrm{ijj}})=C(T_{ij},T_{ij})+\sigma_{\epsilon }^{2}$ for $1\leq j\leq m_{i},\;1\leq i\leq n$. For the second nonparametric model, by assumption, $E(r_{\mathrm{ijk}})=g(|T_{ij}-T_{ik}|)+11_{\left\{T_{ij}=T_{ik}\right\}}\sigma_{\epsilon }^{2}$. Thus, we smooth the data $\left\{(|T_{ij}-T_{ik}|,r_{\mathrm{ijk}}),1\leq j\neq k\leq m_{i},1\leq i\leq n\right\}$ to estimate the function *g*. Specifically, we use univariate *P*-splines,^25^ which approximate *g* using B-spline bases and also control overfit using a smoothness penalty. Then the error variance $\sigma_{\epsilon }^{2}$ can be estimated by the equality $E(r_{\mathrm{ijj}})=g(0)+\sigma_{\epsilon }^{2}$ for $1\leq j\leq m_{i},\;1\leq i\leq n$. Kernel smoothing^26^ could also be used as an alternative to fitting splines.

## 4 Results

### 4.1 Marginal standard charts

**Figure 2.**
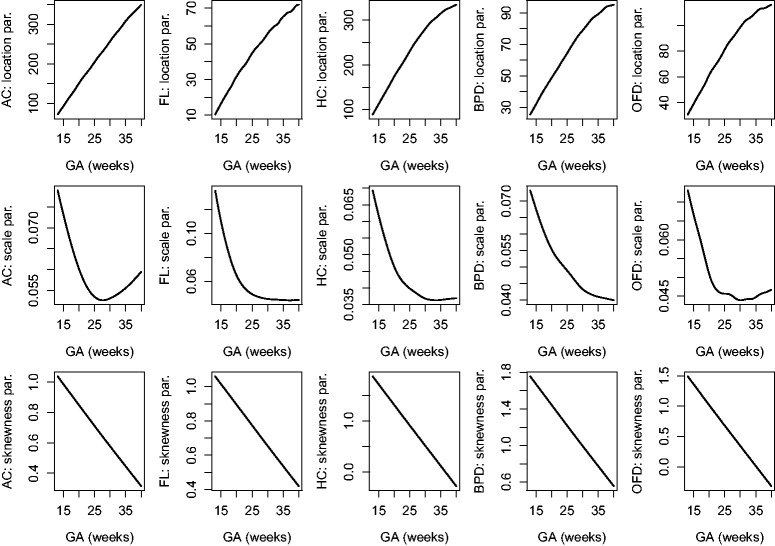
Estimated location, scale and skewness parameters as functions of gestational age for the five fetal growth measurements.

**Figure 3.**
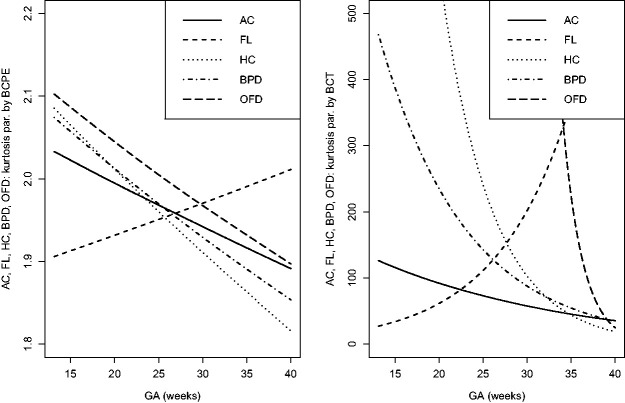
Estimated kurtosis parameters as functions of gestational age for the five fetal growth measurements using BCPE and BCT.

**Figure 4.**
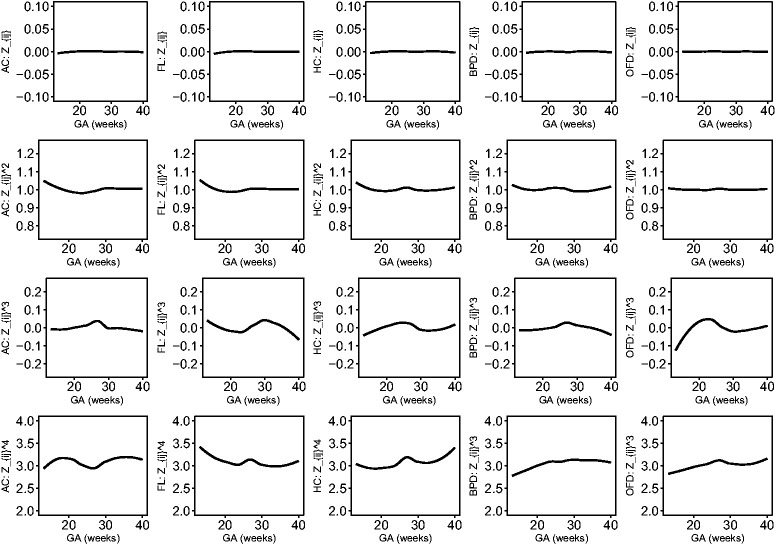
Smooth estimates of the first to fourth moments of the constructed Z-scores for AC, FL, HC, BPD and OFD.

**Figure 5.**
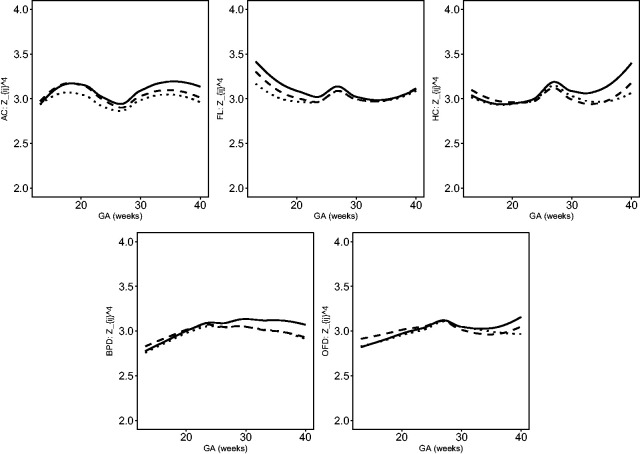
Further comparison of BCCG (solid), BCPE (dashed) and BCT (dotted) on the fourth moments of *Z*-scores.

The estimated location, scale and skewness parameters show that a BCCG transformation model fits the data well (see Figure 2). Our empirical results also indicate that it suffices to use BCCG rather than the more complicated BCPE or BCT, as Figure 3 suggests that the estimated parameter of kurtosis is close to 2 for BCPE model and very large for BCT model. Figure 4 plots the smoothed first to fourth moments of the Z-scores against the gestational age. Specifically, nonparametric smooth functions are fitted to the data $\left\{Z_{ij}^{k},1\leq j\leq m_{i},1\leq i\leq n\right\}$ for k=1,2,3,4. If the Z-scores are indeed marginally normal, then the estimated curves should be close to the respective constant lines *y *=* *0, 1, 0, and 3, respectively. Figure 4 suggests that the BCCG-transformed Z-scores are marginally normally distributed. A closer look at the smoothed fourth moments under different models in Figure 5 confirms that BCPE and BCT are not necessary.

Consequently, the BCCG model will be applied to construct marginal standard charts.

### 4.2 Correlation models

We use the BCCG model to fit the marginal distributions of the raw ultrasound measurements and then convert the transformed measurements into Z-scores. Then different parametric and nonparametric correlation models are compared via model selection criteria: AIC and BIC. Both criteria require the degrees of freedom of the model. For parametric correlation models, it is the number of free parameters. For nonparametric correlation models, the effective degrees of freedom, which evaluates the model complexity of nonparametric smoothers,^27^ will be calculated.

Model comparison results for AC, FL, HC, BPD, and OFD are summarized in Table 3. Table 3 shows that the P1+ model is overall the best model across the three fetal growth measurements. It fits the data best among all parametric models and has a simpler form than all the nonparametric models, and yields the smallest BIC. To quantify the differences among different correlation models, we use P1+ as the reference correlation and evaluate how the other models differ from P1+. Denote $\rho_{jk}^{\text{P1+}}$ the correlation coefficient at times (*j*, *k*) in P1+ correlation matrix, the mean squared error (MSE) of NP1, for example, to P1+ is defined as

$$\text{MSE(NP1,}\;\text{P1+)}=\frac{1}{\left(L-1)(L-2\right)}\sum_{1\leq j<k\leq L}{\left(\rho_{jk}^{\text{NP1}}-\rho_{jk}^{\text{P1+}}\right)}^{2}$$

where *L *=* *183 is the range of gestational age in days in this study.

**Table 3.** Comparison of correlation models.

	AC			FL			HC			BPD			OFD		
Models	-2log-lik	AIC	BIC	-2log-lik	AIC	BIC	-2log-lik	AIC	BIC	-2log-lik	AIC	BIC	-2log-lik	AIC	BIC
P1	4656.31	4660.31	4673.01	2790.43	2794.43	2807.13	1999.78	2003.78	2016.48	1462.85	1466.85	1479.54	4042.71	4046.71	4059.41
P1+	4505.17^a^	4511.17^a^	**4530.21** ^a^	2672.04^a^	**2678.04** ^a^	**2697.08** ^a^	1846.77^a^	1852.77^a^	**1871.82** ^a^	1409.75	1415.75	1434.80	3915.81	3921.81	3940.86
P2	5201.95	5205.95	5218.64	3471.66	3475.66	3488.36	2117.83	2121.83	2134.53	1443.86	1447.86	1460.55	4350.44	4354.44	4367.14
P2+	4540.30	4546.30	4565.34	2708.85	2714.85	2733.89	1943.10	1949.10	1968.15	1334.85	1340.85	1359.90	3979.67	3985.67	4004.72
NP1	**4489.71**	**4509.85**	4573.75	2681.81	2697.74	2748.32	**1743.67**	**1783.82**	1911.26	1286.01	1325.46	1450.69	3785.55	3825.14	3950.79
NP2	4524.09	4530.36	4550.24	**2670.06**	2678.54	2705.47	1863.42	1869.44	1888.52	1415.09	1421.09	1440.14	3929.89	3935.89	3954.93

Note: The bold type denotes the best model in one column. All values are less 40,000 for typographical reasons.

^a^Best parametric model in each column.

Table 4 demonstrates an ignorable difference between P1+ and NP2, as expected because of the stationarity nature of both models. The difference between P1+ and NP1 is small, suggesting that an exponential correlation model with nugget effect is sufficient for fetal growth measurements. Indeed, the average absolute difference in correlation is only 0.020 for AC, 0.021 for FL, 0.025 for HC, 0.031 for BPD, and 0.032 for OFD. The correlations from the other parametric models are relatively more divergent from those of P1+ compared to the nonparametric models NP1 and NP2, indicating that P1+ is superior to other parametric models.

**Table 4.** MSE(·, P1+) comparison.

Model	AC	FL	HC	BPD	OFD
NP1	$3.93\times 10^{-4}$	$4.46\times 10^{-4}$	$6.33\times 10^{-4}$	$9.66\times 10^{-4}$	$1.02\times 10^{-3}$
NP2	$2.25\times 10^{-4}$	$6.46\times 10^{-5}$	$3.75\times 10^{-4}$	$1.30\times 10^{-4}$	$2.53\times 10^{-4}$
P1	$2.87\times 10^{-3}$	$1.94\times 10^{-3}$	$1.98\times 10^{-3}$	$1.42\times 10^{-3}$	$3.42\times 10^{-3}$
P2+	$9.19\times 10^{-4}$	$3.73\times 10^{-4}$	$1.08\times 10^{-3}$	$1.33\times 10^{-3}$	$1.00\times 10^{-3}$
P2	$1.40\times 10^{-2}$	$1.36\times 10^{-2}$	$2.82\times 10^{-3}$	$4.52\times 10^{-4}$	$9.41\times 10^{-3}$

**Figure 6.**
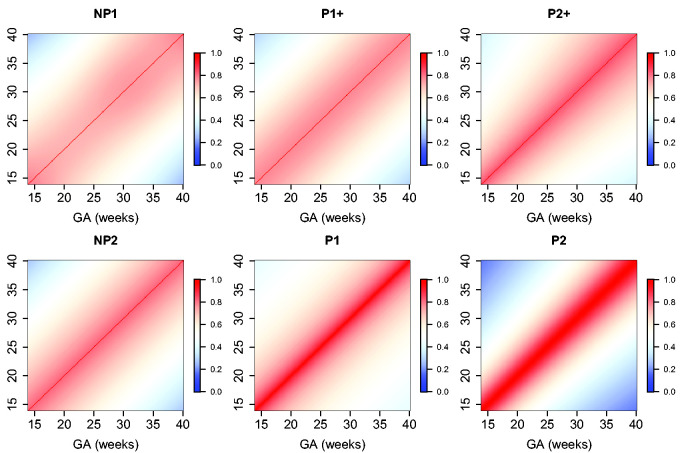
Temporal correlations of standardized AC with different correlation models.

**Figure 7.**
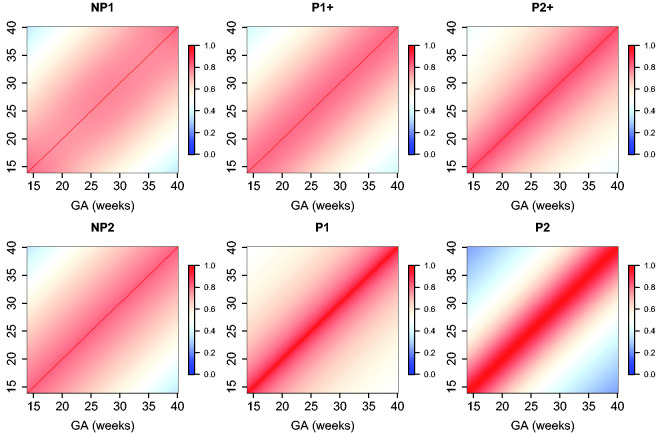
Temporal correlations of standardized FL with different correlation models.

**Figure 8.**
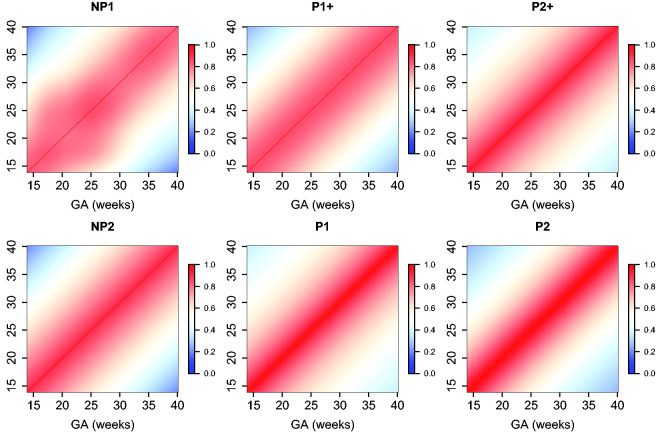
Temporal correlations of standardized HC with different correlation models.

**Figure 9.**
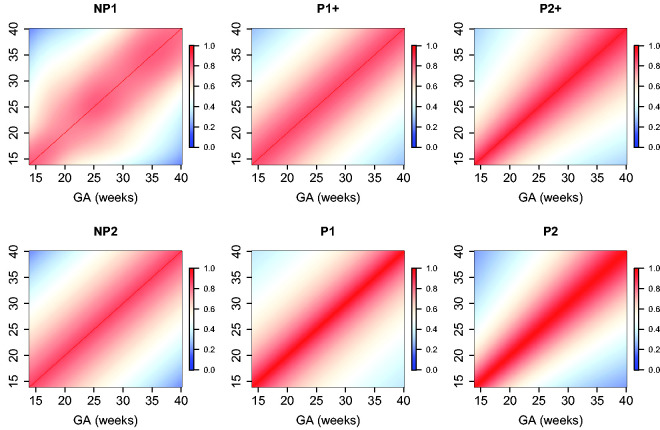
Temporal correlations of standardized BPD with different correlation models.

**Figure 10.**
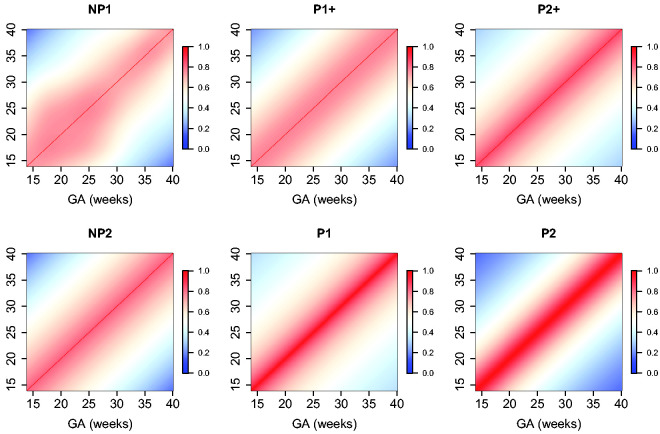
Temporal correlations of standardized OFD with different correlation models.

The estimated parameters for a P1+ model are summarized in Table 5. For illustration, we plot the fitted correlation surface on a grid of gestational age by weeks for AC in Figure 6. Correlation plots for FL, HC, BPD, and OFD are given in Figures 7 to 10.

**Table 5.** Estimated parameters for P1+ correlation models.

Measurement	a	b	$\sigma^{2}$
AC	1.56	0.0060	0.29
FL	1.45	0.0065	0.24
HC	1.54	0.0080	0.17
BPD	1.32	0.0155	0.16
OFD	1.58	0.0075	0.29

**Figure 11.**
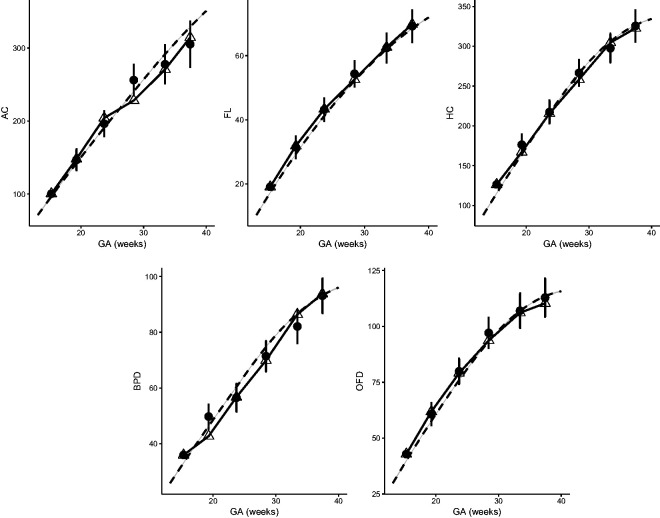
Observed growth trajectory (linked triangles) and predicted measurements (dots) given previous observations of a randomly selected fetus. Dashed line is the population mean.

## 5 Case study: dynamic growth velocity

We study the growth velocity of a randomly selected fetus using the fitted parametric correlation model, whose AC, FL, HC, BPD, and OFD are measured on six occasions between week 15 and week 38. The observed growth trajectories are shown as linked triangles in Figure 11. Based on each observed measurement at *T_j_*, we also dynamically predict the measurement $T_{j+1}$ shown as dots, each with a 95% prediction interval. Specifically, each observed measurement at *T_j_* is transformed to Z-score using equation (2). Then, a conditional Z-score $Z_{〈T_{j+1}|T_{j},\cdots ,T_{1}〉}$ at time $T_{j+1}$ is obtained assuming joint normality with the P1+ correlation model. This conditional Z-score is then transformed back to the original measurement given marginal references. Clinicians might use this approach to compare the observed fetal growth measurements versus its expected measurements at a certain age to assess whether a fetus is growing normally. They can also calculate and compare velocity increments. We will use the correlations studied in this paper for the subsequent clinical paper on conditional fetal velocity for use by clinicians.

For this fetus, selected as random, the growth is regular for FL and HC and can be predicted accurately. For AC, its measurements are higher (still normal) than predicted during the third visit, but much lower than expected during the fourth visit. This suggests that closer monitoring might be needed. The following visits indicate that the AC of the sampled fetus falls consistently below the population mean.

To facilitate the usage of the results in practice, a Shiny application is built along with this paper, where functionalities such as visualization, calculating correlation, prediction and cSDS are integrated for all the five fetal growth measurements (https://lxiao5.shinyapps.io/shinycalculator/). Correlation tables for fetal growth measurements are provided in Tables 6 to 10. The correlations are for weekly intervals, so the results are presented in the form of five 27 x 27 correlation matrices.

## 6 Discussion

We have modelled the correlation function of the fetal growth for transformed HC, BPD, OFD, AC, and FL. Its values are the correlations of two measurements of these five variables made at any time points between 14 and 40 weeks. The FGLS cohort remained healthy with adequate growth and motor development up to 2 years of age, hence making the characterization of the expected correlation of fetal size measurements ideal.^9,11,12,15,28^

The fit of the model for the correlations is adequate.

**Table 6.** Correlation matrix for AC.

Week	14	15	16	17	18	19	20	21	22	23	24	25	26	27	28	29	30	31	32	33	34	35	36	37	38	39	40
14	1.00																										
15	0.77	1.00																									
16	0.76	0.77	1.00																								
17	0.75	0.76	0.77	1.00																							
18	0.74	0.75	0.76	0.77	1.00																						
19	0.72	0.74	0.75	0.76	0.77	1.00																					
20	0.70	0.72	0.74	0.75	0.76	0.77	1.00																				
21	0.69	0.70	0.72	0.74	0.75	0.76	0.77	1.00																			
22	0.67	0.69	0.70	0.72	0.74	0.75	0.76	0.77	1.00																		
23	0.65	0.67	0.69	0.70	0.72	0.74	0.75	0.76	0.77	1.00																	
24	0.63	0.65	0.67	0.69	0.70	0.72	0.74	0.75	0.76	0.77	1.00																
25	0.60	0.63	0.65	0.67	0.69	0.70	0.72	0.74	0.75	0.76	0.77	1.00															
26	0.58	0.60	0.63	0.65	0.67	0.69	0.70	0.72	0.74	0.75	0.76	0.77	1.00														
27	0.56	0.58	0.60	0.63	0.65	0.67	0.69	0.70	0.72	0.74	0.75	0.76	0.77	1.00													
28	0.54	0.56	0.58	0.60	0.63	0.65	0.67	0.69	0.70	0.72	0.74	0.75	0.76	0.77	1.00												
29	0.52	0.54	0.56	0.58	0.60	0.63	0.65	0.67	0.69	0.70	0.72	0.74	0.75	0.76	0.77	1.00											
30	0.50	0.52	0.54	0.56	0.58	0.60	0.63	0.65	0.67	0.69	0.70	0.72	0.74	0.75	0.76	0.77	1.00										
31	0.47	0.50	0.52	0.54	0.56	0.58	0.60	0.63	0.65	0.67	0.69	0.70	0.72	0.74	0.75	0.76	0.77	1.00									
32	0.45	0.47	0.50	0.52	0.54	0.56	0.58	0.60	0.63	0.65	0.67	0.69	0.70	0.72	0.74	0.75	0.76	0.77	1.00								
33	0.43	0.45	0.47	0.50	0.52	0.54	0.56	0.58	0.60	0.63	0.65	0.67	0.69	0.70	0.72	0.74	0.75	0.76	0.77	1.00							
34	0.41	0.43	0.45	0.47	0.50	0.52	0.54	0.56	0.58	0.60	0.63	0.65	0.67	0.69	0.70	0.72	0.74	0.75	0.76	0.77	1.00						
35	0.39	0.41	0.43	0.45	0.47	0.50	0.52	0.54	0.56	0.58	0.60	0.63	0.65	0.67	0.69	0.70	0.72	0.74	0.75	0.76	0.77	1.00					
36	0.37	0.39	0.41	0.43	0.45	0.47	0.50	0.52	0.54	0.56	0.58	0.60	0.63	0.65	0.67	0.69	0.70	0.72	0.74	0.75	0.76	0.77	1.00				
37	0.35	0.37	0.39	0.41	0.43	0.45	0.47	0.50	0.52	0.54	0.56	0.58	0.60	0.63	0.65	0.67	0.69	0.70	0.72	0.74	0.75	0.76	0.77	1.00			
38	0.33	0.35	0.37	0.39	0.41	0.43	0.45	0.47	0.50	0.52	0.54	0.56	0.58	0.60	0.63	0.65	0.67	0.69	0.70	0.72	0.74	0.75	0.76	0.77	1.00		
39	0.32	0.33	0.35	0.37	0.39	0.41	0.43	0.45	0.47	0.50	0.52	0.54	0.56	0.58	0.60	0.63	0.65	0.67	0.69	0.70	0.72	0.74	0.75	0.76	0.77	1.00	
40	0.30	0.32	0.33	0.35	0.37	0.39	0.41	0.43	0.45	0.47	0.50	0.52	0.54	0.56	0.58	0.60	0.63	0.65	0.67	0.69	0.70	0.72	0.74	0.75	0.76	0.77	1.00

**Table 7.** Correlation matrix for FL.

Week	14	15	16	17	18	19	20	21	22	23	24	25	26	27	28	29	30	31	32	33	34	35	36	37	38	39	40
14	1.00																										
15	0.80	1.00																									
16	0.79	0.80	1.00																								
17	0.78	0.79	0.80	1.00																							
18	0.77	0.78	0.79	0.80	1.00																						
19	0.76	0.77	0.78	0.79	0.80	1.00																					
20	0.74	0.76	0.77	0.78	0.79	0.80	1.00																				
21	0.72	0.74	0.76	0.77	0.78	0.79	0.80	1.00																			
22	0.71	0.72	0.74	0.76	0.77	0.78	0.79	0.80	1.00																		
23	0.69	0.71	0.72	0.74	0.76	0.77	0.78	0.79	0.80	1.00																	
24	0.67	0.69	0.71	0.72	0.74	0.76	0.77	0.78	0.79	0.80	1.00																
25	0.66	0.67	0.69	0.71	0.72	0.74	0.76	0.77	0.78	0.79	0.80	1.00															
26	0.64	0.66	0.67	0.69	0.71	0.72	0.74	0.76	0.77	0.78	0.79	0.80	1.00														
27	0.62	0.64	0.66	0.67	0.69	0.71	0.72	0.74	0.76	0.77	0.78	0.79	0.80	1.00													
28	0.60	0.62	0.64	0.66	0.67	0.69	0.71	0.72	0.74	0.76	0.77	0.78	0.79	0.80	1.00												
29	0.58	0.60	0.62	0.64	0.66	0.67	0.69	0.71	0.72	0.74	0.76	0.77	0.78	0.79	0.80	1.00											
30	0.56	0.58	0.60	0.62	0.64	0.66	0.67	0.69	0.71	0.72	0.74	0.76	0.77	0.78	0.79	0.80	1.00										
31	0.55	0.56	0.58	0.60	0.62	0.64	0.66	0.67	0.69	0.71	0.72	0.74	0.76	0.77	0.78	0.79	0.80	1.00									
32	0.53	0.55	0.56	0.58	0.60	0.62	0.64	0.66	0.67	0.69	0.71	0.72	0.74	0.76	0.77	0.78	0.79	0.80	1.00								
33	0.51	0.53	0.55	0.56	0.58	0.60	0.62	0.64	0.66	0.67	0.69	0.71	0.72	0.74	0.76	0.77	0.78	0.79	0.80	1.00							
34	0.49	0.51	0.53	0.55	0.56	0.58	0.60	0.62	0.64	0.66	0.67	0.69	0.71	0.72	0.74	0.76	0.77	0.78	0.79	0.80	1.00						
35	0.47	0.49	0.51	0.53	0.55	0.56	0.58	0.60	0.62	0.64	0.66	0.67	0.69	0.71	0.72	0.74	0.76	0.77	0.78	0.79	0.80	1.00					
36	0.46	0.47	0.49	0.51	0.53	0.55	0.56	0.58	0.60	0.62	0.64	0.66	0.67	0.69	0.71	0.72	0.74	0.76	0.77	0.78	0.79	0.80	1.00				
37	0.44	0.46	0.47	0.49	0.51	0.53	0.55	0.56	0.58	0.60	0.62	0.64	0.66	0.67	0.69	0.71	0.72	0.74	0.76	0.77	0.78	0.79	0.80	1.00			
38	0.42	0.44	0.46	0.47	0.49	0.51	0.53	0.55	0.56	0.58	0.60	0.62	0.64	0.66	0.67	0.69	0.71	0.72	0.74	0.76	0.77	0.78	0.79	0.80	1.00		
39	0.41	0.42	0.44	0.46	0.47	0.49	0.51	0.53	0.55	0.56	0.58	0.60	0.62	0.64	0.66	0.67	0.69	0.71	0.72	0.74	0.76	0.77	0.78	0.79	0.80	1.00	
40	0.39	0.41	0.42	0.44	0.46	0.47	0.49	0.51	0.53	0.55	0.56	0.58	0.60	0.62	0.64	0.66	0.67	0.69	0.71	0.72	0.74	0.76	0.77	0.78	0.79	0.80	1.00

**Table 8.** Correlation matrix for HC.

Week	14	15	16	17	18	19	20	21	22	23	24	25	26	27	28	29	30	31	32	33	34	35	36	37	38	39	40
14	1.00																										
15	0.85	1.00																									
16	0.84	0.85	1.00																								
17	0.82	0.84	0.85	1.00																							
18	0.80	0.82	0.84	0.85	1.00																						
19	0.78	0.80	0.82	0.84	0.85	1.00																					
20	0.76	0.78	0.80	0.82	0.84	0.85	1.00																				
21	0.73	0.76	0.78	0.80	0.82	0.84	0.85	1.00																			
22	0.70	0.73	0.76	0.78	0.80	0.82	0.84	0.85	1.00																		
23	0.68	0.70	0.73	0.76	0.78	0.80	0.82	0.84	0.85	1.00																	
24	0.65	0.68	0.70	0.73	0.76	0.78	0.80	0.82	0.84	0.85	1.00																
25	0.62	0.65	0.68	0.70	0.73	0.76	0.78	0.80	0.82	0.84	0.85	1.00															
26	0.60	0.62	0.65	0.68	0.70	0.73	0.76	0.78	0.80	0.82	0.84	0.85	1.00														
27	0.57	0.60	0.62	0.65	0.68	0.70	0.73	0.76	0.78	0.80	0.82	0.84	0.85	1.00													
28	0.54	0.57	0.60	0.62	0.65	0.68	0.70	0.73	0.76	0.78	0.80	0.82	0.84	0.85	1.00												
29	0.51	0.54	0.57	0.60	0.62	0.65	0.68	0.70	0.73	0.76	0.78	0.80	0.82	0.84	0.85	1.00											
30	0.49	0.51	0.54	0.57	0.60	0.62	0.65	0.68	0.70	0.73	0.76	0.78	0.80	0.82	0.84	0.85	1.00										
31	0.46	0.49	0.51	0.54	0.57	0.60	0.62	0.65	0.68	0.70	0.73	0.76	0.78	0.80	0.82	0.84	0.85	1.00									
32	0.44	0.46	0.49	0.51	0.54	0.57	0.60	0.62	0.65	0.68	0.70	0.73	0.76	0.78	0.80	0.82	0.84	0.85	1.00								
33	0.41	0.44	0.46	0.49	0.51	0.54	0.57	0.60	0.62	0.65	0.68	0.70	0.73	0.76	0.78	0.80	0.82	0.84	0.85	1.00							
34	0.39	0.41	0.44	0.46	0.49	0.51	0.54	0.57	0.60	0.62	0.65	0.68	0.70	0.73	0.76	0.78	0.80	0.82	0.84	0.85	1.00						
35	0.36	0.39	0.41	0.44	0.46	0.49	0.51	0.54	0.57	0.60	0.62	0.65	0.68	0.70	0.73	0.76	0.78	0.80	0.82	0.84	0.85	1.00					
36	0.34	0.36	0.39	0.41	0.44	0.46	0.49	0.51	0.54	0.57	0.60	0.62	0.65	0.68	0.70	0.73	0.76	0.78	0.80	0.82	0.84	0.85	1.00				
37	0.32	0.34	0.36	0.39	0.41	0.44	0.46	0.49	0.51	0.54	0.57	0.60	0.62	0.65	0.68	0.70	0.73	0.76	0.78	0.80	0.82	0.84	0.85	1.00			
38	0.30	0.32	0.34	0.36	0.39	0.41	0.44	0.46	0.49	0.51	0.54	0.57	0.60	0.62	0.65	0.68	0.70	0.73	0.76	0.78	0.80	0.82	0.84	0.85	1.00		
39	0.28	0.30	0.32	0.34	0.36	0.39	0.41	0.44	0.46	0.49	0.51	0.54	0.57	0.60	0.62	0.65	0.68	0.70	0.73	0.76	0.78	0.80	0.82	0.84	0.85	1.00	
40	0.26	0.28	0.30	0.32	0.34	0.36	0.39	0.41	0.44	0.46	0.49	0.51	0.54	0.57	0.60	0.62	0.65	0.68	0.70	0.73	0.76	0.78	0.80	0.82	0.84	0.85	1.00

**Table 9.** Correlation matrix for BPD.

Week	14	15	16	17	18	19	20	21	22	23	24	25	26	27	28	29	30	31	32	33	34	35	36	37	38	39	40
14	1.00																										
15	0.82	1.00																									
16	0.80	0.81	1.00																								
17	0.77	0.79	0.80	1.00																							
18	0.73	0.76	0.78	0.79	1.00																						
19	0.69	0.72	0.75	0.77	0.78	1.00																					
20	0.65	0.69	0.72	0.75	0.76	0.77	1.00																				
21	0.61	0.66	0.69	0.72	0.75	0.76	0.77	1.00																			
22	0.58	0.63	0.67	0.71	0.73	0.75	0.77	0.78	1.00																		
23	0.56	0.61	0.65	0.69	0.72	0.74	0.76	0.77	0.79	1.00																	
24	0.54	0.59	0.64	0.67	0.71	0.73	0.75	0.77	0.79	0.81	1.00																
25	0.52	0.57	0.62	0.66	0.69	0.72	0.74	0.76	0.78	0.81	0.82	1.00															
26	0.51	0.56	0.60	0.64	0.67	0.70	0.72	0.75	0.77	0.80	0.82	0.83	1.00														
27	0.49	0.54	0.59	0.63	0.66	0.68	0.70	0.73	0.76	0.78	0.80	0.82	0.82	1.00													
28	0.47	0.52	0.57	0.61	0.64	0.66	0.68	0.71	0.73	0.76	0.79	0.80	0.81	0.81	1.00												
29	0.45	0.50	0.55	0.59	0.62	0.64	0.66	0.68	0.71	0.74	0.76	0.78	0.80	0.80	0.80	1.00											
30	0.43	0.48	0.53	0.56	0.59	0.61	0.63	0.66	0.68	0.71	0.74	0.76	0.78	0.79	0.79	0.80	1.00										
31	0.41	0.46	0.50	0.54	0.57	0.59	0.61	0.63	0.65	0.68	0.71	0.73	0.75	0.77	0.78	0.79	0.80	1.00									
32	0.39	0.43	0.48	0.51	0.54	0.56	0.58	0.60	0.63	0.65	0.68	0.71	0.73	0.75	0.76	0.78	0.79	0.80	1.00								
33	0.36	0.41	0.45	0.48	0.51	0.53	0.55	0.57	0.60	0.62	0.65	0.68	0.70	0.73	0.75	0.77	0.78	0.80	0.81	1.00							
34	0.34	0.38	0.42	0.46	0.48	0.50	0.52	0.54	0.57	0.59	0.62	0.65	0.68	0.70	0.73	0.75	0.77	0.79	0.81	0.82	1.00						
35	0.32	0.36	0.40	0.43	0.45	0.47	0.49	0.51	0.54	0.56	0.59	0.62	0.65	0.67	0.70	0.73	0.76	0.78	0.80	0.82	0.83	1.00					
36	0.29	0.33	0.37	0.40	0.42	0.44	0.46	0.48	0.50	0.53	0.56	0.59	0.62	0.64	0.67	0.71	0.74	0.76	0.79	0.81	0.82	0.83	1.00				
37	0.27	0.31	0.34	0.37	0.39	0.41	0.43	0.45	0.47	0.49	0.52	0.55	0.58	0.61	0.64	0.68	0.71	0.74	0.77	0.79	0.81	0.82	0.83	1.00			
38	0.24	0.28	0.31	0.34	0.36	0.38	0.40	0.41	0.44	0.46	0.48	0.51	0.54	0.57	0.61	0.64	0.68	0.71	0.74	0.77	0.79	0.81	0.82	0.82	1.00		
39	0.22	0.25	0.28	0.31	0.33	0.35	0.36	0.38	0.40	0.42	0.44	0.47	0.50	0.53	0.57	0.60	0.64	0.68	0.72	0.75	0.77	0.79	0.80	0.81	0.81	1.00	
40	0.20	0.22	0.25	0.28	0.30	0.31	0.33	0.35	0.36	0.38	0.40	0.43	0.46	0.49	0.53	0.57	0.61	0.65	0.68	0.72	0.74	0.77	0.78	0.79	0.80	0.80	1.00

Regression models such as in Ivanescu et al.^29^ may also be used but in general are more difficult to deal with when the data are highly non-normal, as is the case for fetal metrics. The proposed two-stage approach is conceptually simpler, yields easy-to-interpret results, and achieves several aims. First, it gives a marginal standard chart that well handles non-normality of the measurements. Second, the correlation model combined with the marginal standard chart provides a parsimonious approach to prediction and inference at a future visit. Indeed, not only could we predict a future growth given the previous visits (one visit, two visits, etc.) along with a prediction interval, but also we could assess if the current growth is within normal bounds given the previous records.

Although velocity charts could be an important complement to attained size charts,^9^ they are not often used clinically. For example, a clinician may be interested to know whether fetal HC at 20 weeks is a good predictor of that same fetuses HC at 30 weeks. From the correlation between 20 and 30 weeks, we can predict the value of fetal HC at 30 weeks based on its value at 20 weeks. Such prediction can identify fetuses that lag behind in growth.

A limitation of the study is paucity of data and small sample sizes for some pairs of gestational ages especially in early gestation (first trimester) and at term (40 weeks).

In summary, we provide formulae for correlation coefficients for fetal biometry using prospectively collected data in eight countries and diverse settings. They were collected using unified protocols, measurement procedures and standardization. A rigorous data quality process was in place throughout the study. INTERGROWTH-21^st^ Project is the largest prospective study of fetal growth involving multiple measurements per fetus. The correlation coefficients for any pair of data between 14 and 40 weeks and consequently the calculation of a velocity Z-score provide a tool for monitoring fetal growth and development over time. To facilitate this, a web application (Shiny application for now) that calculates the expected correlation between any two time points in the interval 14 to 40 weeks for HC, AC, FL, BPD, and OFD will be made freely available on the INTERGROWTH-21^st^ website where other applications for fetal, preterm, and newborn size are already available (https://intergrowth21.tghn.org/).

Our proposed two-stage approach can be able to accommodate simultaneous modelling of multiple fetal metrics by adapting our two-stage approach. The marginal standard charts can be estimated the same way as the first stage. Then we treat the transformed Z-scores as multiple measurements that are longitudinally observed and model the correlations across measurements and between different times. One option is a nonparametric multivariate functional data analysis.^30^

## Figures and Tables

**Table 10.** Correlation matrix for OFD.

Week	14	15	16	17	18	19	20	21	22	23	24	25	26	27	28	29	30	31	32	33	34	35	36	37	38	39	40
14	1.00																										
15	0.75	1.00																									
16	0.74	0.75	1.00																								
17	0.73	0.75	0.76	1.00																							
18	0.72	0.74	0.76	0.77	1.00																						
19	0.71	0.73	0.75	0.76	0.77	1.00																					
20	0.69	0.72	0.74	0.76	0.76	0.77	1.00																				
21	0.67	0.70	0.73	0.75	0.76	0.76	0.76	1.00																			
22	0.65	0.69	0.72	0.74	0.75	0.76	0.76	0.76	1.00																		
23	0.63	0.67	0.70	0.73	0.74	0.75	0.76	0.76	0.77	1.00																	
24	0.61	0.65	0.69	0.71	0.73	0.74	0.75	0.76	0.76	0.77	1.00																
25	0.59	0.63	0.66	0.69	0.71	0.73	0.74	0.74	0.76	0.76	0.77	1.00															
26	0.56	0.60	0.64	0.67	0.69	0.70	0.72	0.73	0.74	0.75	0.76	0.77	1.00														
27	0.53	0.57	0.61	0.64	0.66	0.68	0.69	0.70	0.72	0.73	0.75	0.75	0.76	1.00													
28	0.50	0.54	0.58	0.61	0.63	0.65	0.66	0.68	0.69	0.71	0.73	0.74	0.74	0.75	1.00												
29	0.47	0.51	0.54	0.57	0.59	0.61	0.62	0.64	0.66	0.68	0.70	0.71	0.72	0.73	0.73	1.00											
30	0.44	0.47	0.50	0.53	0.55	0.57	0.59	0.60	0.63	0.65	0.67	0.69	0.70	0.71	0.72	0.73	1.00										
31	0.40	0.44	0.46	0.49	0.51	0.53	0.55	0.57	0.59	0.61	0.63	0.66	0.67	0.69	0.70	0.72	0.73	1.00									
32	0.37	0.40	0.43	0.45	0.47	0.49	0.51	0.53	0.55	0.58	0.60	0.62	0.65	0.67	0.68	0.70	0.72	0.73	1.00								
33	0.35	0.37	0.40	0.42	0.44	0.46	0.48	0.50	0.52	0.55	0.57	0.60	0.62	0.64	0.66	0.68	0.70	0.72	0.73	1.00							
34	0.32	0.35	0.37	0.39	0.41	0.43	0.45	0.47	0.50	0.52	0.54	0.57	0.59	0.62	0.64	0.66	0.68	0.71	0.72	0.74	1.00						
35	0.30	0.32	0.34	0.37	0.39	0.41	0.43	0.45	0.47	0.50	0.52	0.54	0.57	0.59	0.61	0.64	0.66	0.69	0.71	0.73	0.74	1.00					
36	0.27	0.30	0.32	0.34	0.37	0.39	0.41	0.43	0.45	0.47	0.50	0.52	0.54	0.57	0.59	0.61	0.64	0.66	0.68	0.71	0.73	0.75	1.00				
37	0.25	0.27	0.30	0.32	0.34	0.37	0.39	0.41	0.43	0.45	0.48	0.50	0.52	0.54	0.56	0.59	0.61	0.63	0.66	0.68	0.71	0.73	0.75	1.00			
38	0.22	0.25	0.27	0.30	0.32	0.35	0.37	0.39	0.41	0.43	0.46	0.48	0.50	0.52	0.54	0.56	0.58	0.60	0.62	0.65	0.68	0.71	0.73	0.74	1.00		
39	0.19	0.22	0.25	0.28	0.30	0.32	0.35	0.37	0.39	0.41	0.44	0.46	0.47	0.49	0.51	0.53	0.55	0.57	0.59	0.62	0.65	0.68	0.70	0.72	0.73	1.00	
40	0.17	0.20	0.22	0.25	0.28	0.30	0.32	0.35	0.37	0.39	0.41	0.43	0.45	0.47	0.48	0.50	0.51	0.53	0.55	0.58	0.61	0.64	0.67	0.70	0.72	0.74	1.00
